# Upskilling Rheumatology Occupational Therapists in Work Rehabilitation: An Evaluation of a Job Retention Vocational Rehabilitation Training Course (the Workwell Trial)

**DOI:** 10.1002/msc.70067

**Published:** 2025-02-28

**Authors:** Alison Hammond, Rachel O'Brien, Sarah Woodbridge, Yeliz Prior, Angela Ching, June Culley, Jennifer Parker

**Affiliations:** ^1^ Centre for Human Movement and Rehabilitation School of Health and Society University of Salford Salford UK; ^2^ Versus Arthritis/MRC Centre for Musculoskeletal Health and Work University of Salford Salford UK; ^3^ Occupational Therapy, School of Health Wellbeing & Life Sciences, Sheffield Hallam University Sheffield UK

**Keywords:** arthritis, musculoskeletal, vocational rehabilitation, work, work assessment

## Abstract

**Objectives:**

The objectives were to assess current job retention vocational rehabilitation (JRVR) services for employed individuals with inflammatory arthritis (IA) in rheumatology therapy departments interested in participating in the Workwell trial. Additionally, to modify a JRVR training course to support therapists in delivering JRVR and to evaluate changes in therapists’ knowledge, confidence, and ability following the training.

**Methods:**

This was a mixed‐methods study. Current work services were explored with lead therapists through a cross‐sectional survey about their work rehabilitation service; and one‐to‐one interviews. Feedback from previous course attendees and trainers informed modifications to the training course. Participating therapists completed mailed questionnaires pre‐and post‐training.

**Results:**

Lead therapists from 28 interested departments reported providing JRVR to a median of 7 patients per month (IQR 3–12) for an average of 60 min (IQR 41.25–90). Nine therapists participated in pre‐trial interviews, with themes highlighting variability in referrals, the use of work assessment tools, and advice on ergonomic adjustments. The training course was shortened from three to 2 days by incorporating a pre‐training self‐study pack and reducing lecture time, while increasing practical content such as work assessment demonstrations and extended workshops. Following the training, 32 therapists showed significant improvements in their knowledge and confidence in delivering JRVR (*p* < 0.001).

**Discussion:**

The need for training in work assessment and delivery of complex JRVR was identified. The therapist training course provided was favourably received. Post‐training, therapists’ ability to assess and plan complex JRVR improved.

**Trial Registration:** WORKWELL Trial: ISRCTN: 61762297; Clinical Trials.Gov: NCT03942783

## 1 Background

Around two‐thirds of working people with rheumatoid arthritis (RA) experience presenteeism, or reduced productivity at work, even with low disease activity (Kim, Kaneko, and Takeuchi 2017). Many face work instability as job demands exceed their abilities (Gilworth et al. 2003). RA increases absenteeism, with 36%–84% taking sick leave, and leads to work disability, with 10% stopping work within two years of diagnosis, and 50% within 4.5–22 years, despite modern medication (Gwinnutt et al. 2020). Work‐related impacts account for 39%–86% of the total costs of RA (Hsieh et al. 2020).

Vocational rehabilitation (VR) is provided to individuals of working age with health‐related impairments, limitations, or restrictions with work functioning to optimise work participation (Escorpizo et al. 2011). This can be Return to Work VR (RTWVR) for those on sick leave to enable resuming work. Job Retention (JR) VR is provided to workers experiencing day‐to‐day difficulties performing their job. Providing JRVR could help reduce presenteeism, absenteeism and work disability, thus reducing the costs of RA.

In the UK, many workers with rheumatic and musculoskeletal diseases (RMD) lack access to occupational health support for job retention vocational rehabilitation (JRVR), as 61% of the workforce is employed by small (12.9 million workers) and medium (3.4 million) enterprises (SMEs) (Federation of Small Businesses 2023). SMEs are five times less likely to provide occupational health services than large employers (Department of Work and Pensions & Department of Health and Social Care [DWP & DHSC] 2021). Awareness of rights and responsibilities under the UK Equality Act 2010 and knowledge of appropriate ‘reasonable adjustments’ remain limited among employees and employers (Gov.UK, undated). Only a fifth of workers with RMD have workplace modifications, despite reporting challenges with about a quarter of work activities (Brown et al. 2023). Many deny workplace difficulties, seeing them as a threat to identity (British Society of Rehabilitation Medicine [BSRM] 2021), and delay seeking formal accommodations, relying initially on informal support (Gignac et al. 2011). Self‐management often prioritises physical health and routines over work participation (Valaas et al. 2023). Rheumatology clinics provide an ideal setting to identify workplace challenges and offer JRVR, using tools such as the RA‐Work Instability Scale (RA‐WIS) and UK Workplace Activity Limitations Scale (WALS), which include cut‐offs to guide intervention (Gilworth et al. 2003; Hammond, Tennant, et al. 2023).

JRVR can be stepped care, with progressively complex needs requiring more complex interventions (BSRM 2021):

- Level 1 Brief work advice: for everyone working, especially if developing some work problems, that is, provision of self‐help work advice booklets and online resources, with or without a short discussion of work problems (e.g., up to 30 min). This includes self‐management at work advice, work support available, employment rights under the UK Equality Act, and possible work accommodations (reasonable adjustments), if needed.
- Level 2 Work support: for those with ‘straightforward’ work limitations beginning to impact on work productivity. This includes Level 1 plus, a brief assessment of work problems (e.g., RA‐WIS, WALS), identifying appropriate work accommodations (e.g., equipment, work task modification, flexible hours), disclosing their condition to their manager, asking for work accommodations, signposting to work services, and may include practical activities (e.g., trying equipment). This lasts 30 min to 2 hours, over one or two appointments. Those with good self‐efficacy may only need a single coaching session to manage and negotiate changes at work successfully (BSRM 2021).
- Level 3 Complex intervention: for those with work limitations impacting work ability and work‐life balance, particularly with job strain, and struggling with pain and fatigue at work. This includes a detailed work assessment to identify work problems, an individualised biopsychosocial programme including self‐management at work, physical and psychological interventions, work‐life balance, work accommodations, supporting disclosure to employers and requesting work accommodations, with optional work site visits and/or employer liaison (as required), and enabling other service support (e.g., Access to Work (Gov. UK, undated). This requires more time (e.g., two to 10 h).

VR is provided by occupational therapists who provide Level 1 and 2 JRVR but may need more training to further develop skills (e.g., work assessment, extending knowledge of equipment available, understanding disabled workers’ and employers’ rights and responsibilities, supporting disclosure at work and requesting work accommodations, employer liaison, and developing complex level 3 JRVR interventions) (College of Occupational Therapists 2007; Royal College of Occupational Therapists 2023; Prior and Parnell 2024).

The Workwell trial was a randomised controlled trial of JRVR delivered by National Health Service (NHS) therapists to working people with inflammatory arthritis (IA) (i.e., RA, early IA, psoriatic arthritis) experiencing work problems, and was conducted in UK rheumatology therapy out‐patient clinics (Hammond et al. 2020, 2022). Before trial start, as part of the process evaluation, the following were conducted:

- A survey of JRVR provision in NHS rheumatology therapy departments, expressing an interest in trial participation, followed by interviews with lead participating therapists, to understand the context in which Workwell JRVR would be introduced.
- Modification of an existing JRVR training course (O'Brien et al. 2013) for Workwell trial therapists, to support the fidelity of delivering Workwell JRVR, at Level 2 or 3, depending on individual patients’ needs.
- Evaluation of the modified course, to identify participating therapists’: changes in knowledge of and confidence to deliver JRVR; views about training; and ability to conduct the work assessment included (the UK Work Experience Survey‐Rheumatic Conditions [WES‐RC]) (Hammond et al. 2023a), adapted from the United States WES‐RC (Allaire and Keysor 2009).

## 2 Phase 1: Current Rheumatology Occupational Therapy Work Rehabilitation Services

### 2.1 Background

Three UK surveys investigated VR provision for RMDs in the NHS. Firstly, a national survey of UK occupational therapists working with RMD (predominantly osteoarthritis, RA, and fibromyalgia) in primary, secondary, private, and social care (*n* = 279) identified 92% provided a work service, with splinting, work modifications and pacing being the most common interventions (Coole et al. 2013). Two surveys investigated JRVR provision by rheumatology occupational therapists to working people with IA. In one small survey (*n* = 10), therapists provided Level 2 JRVR to, on average, four employed patients with IA/month, for 45 (inter‐quartile range (IQR) 30–90) minutes. It was usual practice to ask employed patients about work at the initial assessment, as specific work referrals were not received (O'Brien et al. 2013). A national survey (estimated response rate of 63% (78/123) from rheumatology departments) identified that 28% (*n* = 22) of responders provided Level 1 and 72% (*n* = 56) Level 2 VR, including JRVR and RTWVR, to on average six employed patients with IA/month, for 60 (IQR 30–90) minutes/patient (Prior and Hammond 2014). Specific work referrals were rare. A wide variety of individualised interventions and job accommodation recommendations could be provided, according to need (see Box 1). Only 12 used work assessments, usually the RA‐WIS (*n* = 10). The availability and quality of VR varied with experience and service constraints. Most VR was provided by occupational therapists, with only five departments stating that physiotherapy also did so. A recent short survey of rheumatology physiotherapists identified that around 50% of patients provide RTWVR but did not ask about JRVR, or what VR was provided (Gregory, Burchett, and McCrum 2021). No surveys could be identified about VR provision by rheumatology nurses.

**BOX 1 Summary of rheumatology occupational therapists’ work‐related service provision.**

1

**Table:** 

Work intervention provided	% therapists providing
Fatigue management for work	100%
Splinting	98%
Ergonomic modifications (i.e. postural advice, work positioning, joint protection, task rotation)	91%
Alternative equipment recommendations	88%
Relaxation/stress management	86%
Workstation modifications	84%
Recommending changes to work duties; work shift patterns	84%
Exercise for work	75%
Supported (graded) return to work after sick leave	71%
Enabling access in the workplace	61%
Liaison with employers: Occupational health, line managers	50%
Supporting disclosure	41%
Conducting work site visits	28%
[Prior and Hammond 2014]

The aims of Phase 1 were to investigate usual JRVR provision in rheumatology therapy departments (a) by surveying lead therapists expressing interest in Workwell trial participation; and (b) to interview lead therapists in participating departments.

### 2.2 Method

#### 2.2.1 Design, Recruitment and Participants

Following ethical approval from the University of Salford Ethical Approval Panel (HSR1819‐010), invitations for trial participation were circulated to members of the Royal College of Occupational Therapists Rheumatology Forum; rheumatology physiotherapy networks in the North‐West and Midlands English regions; four Comprehensive Local Research Networks (which support research regionally in the NHS) expressing interest in supporting trial recruitment; and leafleting at a British Society of Rheumatology (BSR) conference. These contacts were asked to send snowball invitations to other rheumatology therapy colleagues nationally. A cross‐sectional survey was conducted by e‐mail with the lead responding therapist at departments expressing interest. Survey completion was indicative of implied consent. Lead therapists from participating departments were invited to participate in interviews following informed, written consent.

#### 2.2.2 Data Collection

Survey content was derived from previous surveys (O'Brien et al. 2013; Prior and Hammond 2014). This included: if rheumatology clinics refer employed patients with IA for VR; levels of JRVR provided; estimated numbers of working patients with IA providing each level to per month; how long for; and work assessments used. Three types of VR were defined as above (levels 1 to 3). The interview schedule was devised by the study team focusing on JRVR provision.

#### 2.2.3 Data Analyses

The survey data were analysed descriptively, with frequencies and medians (IQR), as not normally distributed. Differences between trial participating versus non‐participating departments were analysed using Mann–Whitney U Tests (Statistical Package for the Social Sciences (SPSS) v26 (IBM Corp 2019). The interview data were analysed using an inductive reflexive thematic analysis (Braun and Clarke 2019, 2021).

### 2.3 Results

#### 2.3.1 Survey Results

Eighteen of 28 departments expressing interest continued to participate. Reasons for the 10 not doing so (including all six with interested physiotherapists) included reduced staffing levels, and lack of clarity about trial excess treatment cost payments to NHS Trusts via the National Institutes of Health Research. Nine (of 18) lead occupational therapists were interviewed about JRVR provision.

Most departments received referrals from rheumatology clinics for work issues. All departments reported they routinely asked employed patients with IA about work issues, irrespective of the reason for referral. On average, departments treated 7 (IQR 3–12) employed patients with IA/month for work issues, totalling 226/month across all 28 departments. Of these, 18.5% (*n* = 42) received Level 1 JRVR; 77% (*n* = 174) Level 2; and 4.5% (*n* = 10) Level 3. The most common form, Level 2, was provided for an average of 60 (IQR 41.25–90.00) minutes/patient. There were no differences between participating and non‐participating departments in terms of type of service provided, number of patients receiving each, and duration of interventions (Table 1).

**TABLE 1:** Work provision at participating and non‐participating rheumatology departments in the Workwell trial (*n* = 28).

	Participating departments (*n* = 18)	Non‐participating departments (*n* = 10)	Total (*n* = 28)	*p*
Location:				
England	15	7	22	—
Wales	2	0	2	
Scotland	1	2	3	
Northern Ireland	0	1	1	
No. of therapists able to support trial at sites:				
1	5	4	9	
2	6	4	10	
3	7	2	9	
Receive work referrals from clinic (yes)	15	10	25	0.22^a^
No. employed patients treated for work issues/month, median (IQR)	8.00	6.00	6.00	0.61
	(2.50, 12.00)	(2.75, 12.75)	(2.50, 12.00)	
Level 1 JRVR: Brief work advice (< 30 min)				
No. departments providing, *n*	7	5	12	0.51^a^
No. employed patients treated/month, median (IQR)	0	1	3.00	0.81
	(0, 2.25)	(0, 3.50)	(2.00, 5.00)	
Duration (minutes), median (IQR)	20.00	15.00	17.50	1.00
	(10.00,30.00)	(10.00,30.00)	(10.00, 30.00)	
Level 2 JRVR: Work advice (30–< 120 min)				
No. departments providing, *n*	17	10	28	0.62^a^
No. employed patients treated/month, median (IQR)	6.00	4.00	5.00	0.49
	(2.00, 10.25)	(2.00, 10.00)	(2.00, 10.00)	
Duration (minutes), median (IQR)	60.00	52.50	60.00	0.52
	(42.50,105.00)	(30.00, 86.25)	(41.25, 90.00)	
Level 3: JRVR complex intervention (120 min–10 h)				
No. departments providing, *n*	4	2	6	0.55^a^
No. employed patients treated/month, median (IQR)	0	0	1.00	0.71
	(0, 0.50)	(0, 0.75)	(0.20, 2.00)	
Duration (minutes), median	105.00^b^	135.00	120.00	0.40
(IQR)	(67.50,142.50)	(120.00,150.00)	(90.00, 150.00)	

*Note:* Mann–Whitney U tests conducted unless denoted as.

^a^ Chi‐square (df, 1).

^b^ one department described service provision lasting less than 120 min as ‘Work Rehabilitation’.

Only those departments providing Level 3 VR used work assessments (*n* = 9): the RA‐WIS (*n* = 4); the WES‐RC (*n* = 2); their own department assessment (*n* = 2); the Allied Health Professions (AHP) Health and Work Report (Allied Health Professions Federation 2019)) (*n* = 1); an adapted version of the ‘Americans with Disabilities Act Work Site Assessment’ (*n* = 1); or a Visual Display Unit Checklist (*n* = 1).

#### 2.3.2 Interview Findings

Nine therapists from England and Scotland participated in pre‐trial interviews between November 2019 and February 2020, which revealed key themes around variability in referrals, the use of work assessment tools, and ergonomic advice. Interviews indicated Level 1 and 2 JRVR were normally provided but interventions were limited by staffing constraints, staff training and experience. All wanted to expand their work provision. Therapists found the JRVR training comprehensive and valuable, distinguishing it from their usual work advice practices. They noted potential benefits for patients, employers, and rheumatology services. Overall, the training boosted their confidence and competence in delivering JRVR, but they had concerns about staffing constraints (Supporting Information S1: File 1).

### 2.4 Discussion

Unlike earlier surveys, where referrals from rheumatology clinics for work issues were rare (O'Brien et al. 2013; Prior and Hammond 2014), almost all therapy departments now receive these, and therapists routinely identify work needs of those referred for other reasons. In the intervening years, the Royal College of Occupational Therapists (RCOT), in partnership with Public Health England (PHE), has trained over 100 occupational therapists and healthcare professionals as Health and Work Champions (RCOT 2020) and the BSR promoted rheumatology teams asking, ‘the work question’ (BSR 2022). Additionally, the European Alliance of Associations for Rheumatology (EULAR) launched the Time2Work campaign to mark World Arthritis Day in 2019 (EULAR 2019). These awareness campaigns may have led to some increase in work‐related service provision, although the level of JRVR provided remained up to level 2. Limitations were that we could only interview half of the lead participating therapists. The survey and interviews indicated that therapists needed further training to deliver the Workwell JRVR programme, particularly at level 3.

## 3 Phase 2: Workwell Therapist Training Course Modification

### 3.1 Background

The Workwell JRVR programme is designed for working people with IA experiencing work activity limitations, scoring ≥ 10 on the RA‐WIS. Gilworth et al. (2003) identified 80% of those scoring 10–17 (moderate work instability), and 95% of those scoring > 17 (high work instability), will need work modifications. Accordingly, therapists should be able to provide Level 3 JRVR, including conducting a semi‐structured work assessment (e.g., the UK WES‐RC), devising an appropriate biopsychosocial VR plan, and using behavioural change skills to enable working people with IA to make changes in the workplace (Hammond et al. 2022). The Workwell JRVR programme is summarised in Supporting Information S1: File 2.

There is some post‐graduate training in JRVR available to health professionals, for example, a few generic: Masters’ level modules in VR, short courses provided by members of the Vocational Rehabilitation Association (VRA) (VRA 2024), and online learning to support health professionals in VR (NHS England e‐learning for Healthcare Hub 2025). At the time of this study, no short courses were available for health professionals on JRVR focused on RMD.

### 3.2 Modifying the Workwell Therapist Training Course

We initially developed a course to enhance therapists’ JRVR skills for our feasibility trial (O'Brien et al. 2013; Hammond et al. 2017). This lasted 3‐days including:

- Work assessment to collaboratively identify and prioritise work problems to be addressed in VR, using the UK WES‐RC.
- Ergonomic adaptation of work activities, to better fit the person’s abilities to their job demands
- Work Environment adaptations including seating, adapted tools and workstations.
- Work accommodations, or ‘reasonable adjustments’ under the UK Equality Act 2010.

Skills that occupational therapists regularly use with IA patients (e.g., splinting, joint protection, fatigue management, work‐life balance) did not need to be included, as therapists could readily integrate these into the Workwell JRVR programme.

The revised training course was designed to mirror the typical service user’s journey through JRVR. Informed by the principles of a heutagogical approach, which empowered therapists to take greater ownership of their learning, fostering autonomy and critical reflection. This aimed to improve their confidence and adaptability in delivering complex JRVR interventions. The course developers—comprising a rheumatology occupational therapist with expertise in JRVR, an ergonomics consultant, academic learning specialists, and patient research partners—reviewed feedback from therapists and trainers during the feasibility trial, with insights from therapist interviews (Prior et al. 2015) and made changes to better align the course with real‐world practice (see Box 2).

**BOX 2 Updating the Workwell therapist training course**

1

**Table:** 

Feedback from therapists and trainers following the feasibility trial training	Changes made for the Workwell trial training course
Course 3 days (i.e., 2 days plus 1 day follow‐up): Therapists liked duration but found it difficult to obtain more than 2 days from work to attend training.	Reduced to 2 days. Developed a pre‐course self‐study pack to enable some content to be addressed in advance.
WES‐RC:	
Opportunity to see WES‐RC being conducted. Therapists unfamiliar with conducting semi‐structured interviews with standardised assessments.	Introduction to WES‐RC and Workwell Solutions Manual included. 30‐min role play by two experienced VR therapists/trainers demonstrating how to perform WES‐RC using ‘conversational’ approach. Followed by discussion and demonstration: how to prioritise work problems and collaboratively formulate solutions.
Case study group work using WES‐RC was valuable; increase the group size (from two therapists); increase the number of cases; and an opportunity to practice WES‐RC during the course.	Group size increased to three or four. Additional case studies. Case studies were re‐timetabled to occur immediately after WES‐RC demonstration, with role play integrated into case study groupwork. Members took turns to be therapist/client/observer (5 min each including peer feedback) to identify work barriers and formulate and prioritise problems.
The Workwell Solutions Manual was valued and used consistently during treatment with feasibility trial participants. Considered as ‘the Bible’	Workwell Solutions Manual was updated. Re‐structured to clearly follow the structure of the WES‐RC. All therapists were given a copy.
	Online version made available during trial.
Clearer idea how to fill in a WES‐RC, write notes related to sections, write a plan and record treatment notes/action plans.	Sample WES‐RC and treatment notes were provided to all therapists.
	Standard Operating Procedures written describing WES‐RC documentation and treatment procedures (copy for each therapist).
Practical workshops	
Reduce taught content.	Reduced lectures from 4.4 to 2.15 h. Removed lecture by Disabled Employment Advisor, as now rarely involved in JRVR.
	Introductory reading material on ergonomics in pre‐training self‐study to compensate for some taught content (Berg‐Rice 2008). Changed some lecture content into practical workshops.
Increase practical content. (Previously five practical workshops, each 20–30 min).	Increased duration of the five workshops to 45–60 min each.
Increase time on seating workshop	Workshop time increased. A sales representative from a seating company brought in six models of chairs, which therapists tried, adjusted, and identified which models suitable for which uses.
More on computer workstations and equipment	A wider variety of computer equipment (mice, keyboards) was available to try and evaluate. Increased content in the Workwell Solutions Manual on equipment.
Input from patient partners with RA	Two patient partners discussed the lived experience of working with RA, issues liaising with employers, and different reactions of employers and co‐workers
More time on legislation, employer liaison and disclosure	Patient partners discussed disclosure issues, liaison with Human Resources, and rights under the Equality Act 2010. Increased time in the practical workshop from 20 to 60 min to include writing summary letters for patients to provide to employers (if required). Key feedback on sample letters.
Self‐study	
Post‐training study too long: previously included also conducting work analysis using the Ergonomic Assessment Tool for Arthritis (EATA: Backman Village and Lacaille 2008)	EATA removed from course/JRVR as therapists reported lacked time in study and practice to complete. One structured assessment (WES‐RC) was enough to complete.
Some therapists lacked confidence in analysing work‐related activities	Pre‐training self‐study: refresher reading on activity analysis, grading, and adapting (Thomas 2015); activity analysis of work activities (selected YouTube videos). Feedback and discussion of activity analysis on day 1. Activity analysis (physical, psychological, social) integrated within practical workshops. Increased range of tools available for use and analysis.
Some therapists wanted to be able to observe a wider variety of jobs/have factory visits before training.	Optional pre‐training self‐study: watch YouTube channels and TV programmes showing different jobs and workplaces (example programmes given).
Too much self‐study	Reduced and re‐structured into ‘bite‐size’ pieces.
Mock WES‐RC	
More input was wanted on what expected to do	Additional session in programme explaining task. Example of a completed WES‐RC and treatment plan provided to all therapists. Opportunity in case studies for brief role play of therapist or patient role, with peer feedback.

Abbreviation: WES‐RC, Work Experience Survey‐Rheumatic Conditions.

To reduce the course content to 2‐days, a short pre‐course self‐study pack was developed and provided 2 months prior to the training course, allowing time for completion. This included: a book chapter about VR (Berg‐Rice 2008) and one on activity analysis (Thomas 2015); watching two YouTube videos of people working and then conducting work‐related activity analyses for these; and the background and treatment sections of the Workwell trial protocol. For those less familiar with different work environments and jobs, optional suggestions included watching selected TV programmes, and YouTube channels focusing on work. The Workwell training course self‐study and course content are in Supporting Information S1: File 3.

Three training courses were delivered over a 2‐month period, with therapists choosing which to join. On day one of the training courses, therapists were given a folder of materials for each session. Following the course introduction and summary of the WORKWELL trial, timescales and therapists’ role, they were introduced to the Work Solutions Manual, containing detailed solutions for various work problems (linked to sections of the WES‐RC) for reference when delivering JRVR. Each therapist was given a hard and electronic copy of this manual, the UK WES‐RC and UK WES‐RC Manual (Hammond et al. 2023a, 2023b), and a completed WES‐RC with VR plan and treatment notes from a case study. The Work Solutions Manual table of contents is in Supporting Information S1: File 4. The WES‐RC was then introduced and the trainers role‐played conducting the WES‐RC, demonstrating a conversational approach and moving quickly through sections irrelevant to the patient (e.g., physical activities not required in the patient’s job; manager relationships if self‐employed). Therapists then worked in four small groups on one case study per group, using the WES‐RC and the Workwell Solutions Manual and pooling their knowledge, to identify work problems and possible solutions. Four different case studies were used (a street cleaner/road worker, primary school kitchen assistant, design engineer (administrative position), and warehouse operative). Each group presented their findings to the whole group. Following this, activity analyses from the pre‐course self‐study were discussed. The day ended with an explanation and discussion of the post‐training telephone mock WES‐RC (see below).

Day two included five practical workshops including: workstation assessment and seating, upper limb strategies and tool use, work environment and clothing, load handling, disclosure, employer liaison, and relevant legislation (the Equality Act 2010 [Legislation.gov.uk, a] and the Health & Safety at Work Act 1974 [Legislation.gov.uk, b]). Therapists analysed equipment and strategies in relation to potential work problems. Two patient partners then shared their journey following diagnosis, their work difficulties and coping strategies. The final session revisited the case studies on day one, reviewing problems identified and solutions considered, taking into account the knowledge therapists gained from the workshops.

Following the training course, therapists completed a telephone mock WES‐RC with a trainer (RO'B or SW) role‐playing one of two case studies provided in advance (a nursery manager or a convenience store manager, both of whom experienced physical limitations and had psychological changes). Therapists then completed the prioritised problems and proposed treatment plan section of the WES‐RC. Therapists sent their completed WES‐RC to the two trainers, who identified each therapist’s ability to assess for and plan appropriate JRVR. If trainers had concerns about the WES‐RC interview and/or proposed treatment plan, therapists were asked to re‐do the mock WES‐RC with a trainer using another case study.

Once therapists started treating trial participants, two Workwell JRVR trainers (RO'B or SW) acted as mentors. Therapists could contact them by e‐mail or telephone for advice on assessment and treatment for any participant. Group meetings were scheduled each month, at varying times of the day, which therapists could join to share questions and offer solutions about participants’ work‐based problems. An e‐mail discussion group was also set up for the same purpose. Therapists were requested to share their second participant’s WES‐RC (anonymised) with a mentor following the participant’s second appointment. Mentors reviewed content in terms of coherence of problems identified, treatment plans and solutions being provided, and gave feedback via e‐mail, telephone or video call, with additional advice as necessary. The second participant was recommended to allow therapists to become familiar with the Workwell process.

## 4 Phase 3: Evaluation of the Workwell Therapist Training Course

### 4.1 Background

An important component of trials of complex interventions is a theory‐driven process evaluation to measure what was delivered. A conceptual framework for implementation fidelity (CFIF) identifies that the degree of fidelity is influenced by moderating factors, such as facilitation strategies to enable uniform delivery (e.g., training, manuals, feedback to therapists on delivery) (Carroll et al. 2007). The aim of this study was to evaluate therapists’ perceived ability to deliver JRVR pre‐and post‐training, their views of the course and support materials to support future development, and trainers’ views of therapists’ competency to assess and plan complex JRVR.

### 4.2 Method

#### 4.2.1 Design, Recruitment and Participants

Following informed, written consent, a longitudinal survey was conducted pre‐ and post‐training. Training was mandatory to deliver JRVR in the trial. All participating therapists were invited to participate.

#### 4.2.2 Data Collection

Pre‐training, therapists completed a mailed questionnaire and returned it on day one of the course. This included demographic information (profession, job grade, years of work experience) and perceptions of their knowledge of and confidence to provide JRVR (0 = very limited to 4 = excellent). The Evidence Based Practice Attitudes Scale (EBPAS) was included to evaluate the willingness to adopt evidence‐based practices (Aarons 2004). This consists of 15 items in four sub‐scales: Requirements (i.e., required to provide interventions in a specified way by manager, employer and/or state; three items); Appeal (i.e., the intuitive appeal of an intervention which makes sense: 4 items); Openness (i.e., to new interventions and adopting change: 4 items); and Divergence (i.e., perceived difference between current and new practices: 4 items). Questions are asked about ‘new types of interventions, treatments and therapies’ in general. It was specified that these included Workwell JRVRs. Participants indicated agreement with statements as 0 = not at all, to 4 = to a very great extent. An average EBPAS score can also be created (Supporting Information S1: File 5).

Post‐training, the same measures were collected. Therapists were also asked about the relevance of training course components, on a scale of 0 = not at all relevant, to 4 = extremely relevant. Additionally, they reported their views, in free‐text boxes on three course components they gained the most from, three not considered useful, how to improve the course, if it could be delivered differently, any further practical activities required, and if so what, if the time allocated to training (i.e., pre‐course study; training course; mock WES‐RC interview) was appropriate, and preferred duration of training. This was mailed to participants after their mock WES‐RC assessment was submitted (Supporting Information S1: File 6).

The trainers devised a form to assess therapists’ telephone mock WES‐RC, and to support formally mentoring therapists with their second participant. Therapists’ abilities were identified as good, satisfactory, or poor. Trainers discussed the mock WES‐RC assessments deciding on overall ability and the feedback to provide (Supporting Information S1: File 7).

#### 4.2.3 Data Analysis

Demographic data were summarised appropriately, with means (standard deviations) for experience duration, and medians (IQR) for VR and EBPAS scores. Differences pre‐and post‐training were analysed using Wilcoxon tests (SPSS v26 (IBM Corp 2019)). Qualitative data were analysed using content analysis, as answers were generally brief. Two researchers independently analysed data and agreed on the results (Bengtsson 2016).

### 4.3 Results

Courses were attended by 10–16 therapists each. Of the 38 therapists attending the training, 36 consented and completed the pre‐training questionnaire. Of these, 32 returned the post‐training questionnaire. All were occupational therapists: 30 women and two men; Band 5 *n* = 2; Band 6 *n* = 15; Band 7 *n* = 11; and Band 8 *n* = 4. Seven held a diploma in Occupational Therapy, 24 a degree and one pre‐registration MSc. Six additionally held post‐graduate qualifications (certificate, diploma, or M.Sc. module(s)/degree). Duration working as a therapist was 18.1 (SD 7.5) years, of which 11 (SD 6.7) years were in rheumatology. Results were analysed for the 32 returning pre‐ and post‐training questionnaires.

Knowledge of and confidence in delivering work rehabilitation increased significantly post‐training Scores on the EBPAS were already high at baseline, with most considering EBP very relevant. Openness sub‐scale scores increased significantly post‐training (*p* = 0.04) (Table 2).

**TABLE 2:** Work rehabilitation and Evidence Based Practice Knowledge and confidence in providing work rehabilitation pre‐ and post‐training (*n* = 32).

	Pre‐training (*n* = 32)	Post‐training (*n* = 32)	*p*
Knowledge:			
work rehabilitation	1.00 (1.00, 2.00)	3.00 (2.00, 3.00)	< 0.001
work rehabilitation process	1.00 (1.00, 2.00)	3.00 (2.00, 3.00)	< 0.001
work rehabilitation strategies	1.00 (1.00–2.00)	3.00 (2.00–3.00)	< 0.001
relevant legislation and policy	1.00 (1.00–1.75)	2.00 (2.00–2.75)	< 0.001
Confidence:			
Completing a work assessment	1.00 (1.00, 2.00)	3.00 (2.00–3.00)	< 0.001
Identifying appropriate work solutions and strategies	2.00 (1.00–2.00)	3.00 (2.00–3.00)	< 0.001
Evidence Based Practice Attitudes Scale:			
Requirements (0–4)	3.00 (2.00–3.75)	3.00 (2.00–3.00)	0.57
Appeal (0–4)	3.00 (3.00–3.50)	3.00 (3.00–3.50)	0.98
Openness (0–4)	3.00 (2.00–3.00)	3.00 (3.00–3.00)	0.04
Divergence (0–4)	3.50 (3.00–4.00)	3.50 (3.13–4.00)	0.11
Total (0–4)	3.00 (3.00–3.00)	3.00 (3.00–3.00)	0.26

All training components were considered very or extremely relevant by at least two‐thirds of the respondents. The most relevant were observing the WES‐RC being conducted; case studies applying the WES‐RC (including identifying key barriers and appropriate solutions); conducting a mock telephone WES‐RC with a trainer; and practical workshops, particularly on employment rights, disclosure, and employer liaison (including writing reports for patients) (Table 3).

**TABLE 3:** Relevance of different components of the Workwell training course (*n* = 32).

Score range 0–4	Programme time (mins):	Median (IQR)	Very/extremely relevant (n)
Pre‐training self‐study pack:			
Ergonomics and therapy: An introduction (Berg Rice 2008)	60^a^	3.00 (2.00–3.00)	23
Activity analysis (Thomas 2015)	60^a^	3.00 (2.00–3.00)	22
Activity analyses *x* 2 (YouTube videos)	120^a^	3.00 (3.00–3.00)	26
Workwell protocol: Background and treatment sections	60^a^	4.00 (3.00–4.00)	30
Training course day 1:			
Introduction to Workwell trial	40	4.00 (3.00–4.00)	31
Introduction to conducting WES‐RC and using Workwell Solutions Manual (including 30‐min demonstration of WES‐RC interview by experienced VR therapists)	60	4.00 (3.00–4.00)	31
Case studies with the WES‐RC (4 cases: Small group work)	90	4.00 (3.00–4.00)	31
Group case study feedback *x* 4: work barriers and solutions	85	3.00 (3.00–4.00)	30
Review of pre‐training study: Activity analysis (YouTube videos)	30	3.00 (2.25–3.75)	24
Question time: Activity analysis, work solutions	25	3.00 (3.00–4.00)	27
Introduction to mock telephone WES‐RC activity with your mentor	15	3.00 (3.00–4.00)	29
Training course day 2:			
Workshop 1: Workstation assessment and seating	45	3.50 (3.00–4.00)	28
Workshop 2: Upper limb strategies and Tool use	45	3.00 (3.00–4.00)	28
Workshop 3: Environment influences and protective clothing	45	3.00 (3.00–4.00)	29
Workshop 4: Load handling	45	3.00 (3.00–4.00)	28
Workshop 5: Disclosure, Rights and	60	3.50 (3.00–4.00)	30
Employer liaison			
Discussion: (Patient partners): working with RA; and Human Resources policy/practices issues	30	3.00 (3.00–4.00)	31
Case studies review: Using	60	3.00 (3.00–4.00)	30
Workwell Solutions Manual and identifying Solutions			
Workwell trial: Avoiding control group contamination & what you can do with the control group	20	4.00 (3.00–4.00)	30
Post‐training: Mock‐WES‐RC			
Telephone mock WES‐RC with mentor	75	4.00 (3.00–4.00)	26
Completing treatment plan:			
• prioritising problems	60^a^	3.00 (3.00–4.00)	25
• planning your intervention	60^a^	3.00 (3.00–4.00)	26
Feedback on telephone WES‐RC	30	3.00 (3.00–4.00)	25

Abbreviation: WES‐RC, Work Experience Survey‐Rheumatic Conditions.

^a^ guide times.

The free text responses supported this. Of the 90 comments (*n* = 32 therapists) about the most beneficial course elements, over half related to:

1. Learning how to use the WES‐RC: observing the WES‐RC being conducted, its use in case studies and conducting a mock WES‐RC interview. For example, ‘*observing the WES‐RC using a more relaxed approach to the assessment form*’ [ID1] and ‘*demystifying the WES‐RC and permission for the assessment to be a natural conversation*’ [ID15].
2. The practical workshops, with time discussing practical problems and jointly discussing solutions, were particularly commented on (Table 4).

**TABLE 4:** More and less useful Workwell training components (*n* = 32).

	Most useful		Less useful
	(*n* = 90)^a^		(*n* = 29)^a^
All training useful	9		—
**The WES‐RC**	**33**		**9**
Demonstrating an interview being conducted	12	Demonstration could be shorter	1
Learning how to complete the WES‐RC in case studies and discussion	8	Needed more information in case studies	
Conducting the mock telephone WES‐RC with mentor	13	Needed more information about mock WES‐RC	1
		Length of time between training and mock WES‐RC	4
**Practical workshops**	**19**		**8**
Workshops and group discussions assisted finding solutions	15	More teaching, less workshops	2
Seating	4	Seating	2
		Protective clothing/hand tools needed more solutions	3
		Mostly core OT skills	1
**Learning experiences**	**8**		**3**
Group learning/discussion	6	Some repetition of pre‐	1
		study materials	
Reassurance that not always solutions	1	Some session learning outcomes unclear	1
Learning from experienced work rehabilitation OTs	1	Difficult room environment	1
**Pre‐course study on activity**	**7**		**7**
**analysis/YouTube videos of**			
**people working**			
Analysing YouTube videos helpful	6	More discussion of analysing YouTube videos needed	6
Set reading	1	Set reading	1
**Rights/Legislation, writing**	**7**	Less time writing	**1**
**letters/reports (for patient/**		letters/reports	
**employer) and disclosure**			
**Using the Workwell Solutions**	**4**		‐
**Manual**			
**Patient partners: working with**	**3**		**1**
**RA/HR issues**			

*Note:* **numbers in bold** represent number of therapists commenting on each topic. Participants could make more than one comment per topic.

Abbreviation: WES‐RC, Work Experience Survey‐Rheumatic Conditions.

^a^ total number of comments made.

Of the 29 comments about what was least useful (*n* = 21 therapists), most of these focused on wanting either more information about or time spent on (*n* = 12), or less time on (*n* = 10), specific components in the training course. The most frequent course improvements and additional activities were to have videos of the WES‐RC being conducted with a variety of clients for future reference after the course (*n* = 7); more time on employment legislation, reasonable adjustments and disclosure (*n* = 6); more input on computer workstation adaptations/equipment (*n* = 5); and more examples of completed WES‐RC with treatments notes (*n* = 3). Most thought the amount of time allocated for training (i.e., approximately 4 days overall, including pre‐course study and the post‐course WES‐RC activity) was about right (*n* = 23). However, time for pre‐course self‐study was considered not enough (*n* = 5), could have been less (*n* = 1) and a lot to expect (*n* = 1). The 2 days in‐person training was about right (*n* = 16), although five wanted longer (three or 4 days). The opportunity to meet in‐person with other therapists to share experiences was valued. Five commented that the time between training and their being able to complete the mock‐WES‐RC assessment was too long, as a result of problems identifying times when both the therapist and trainer were available (Table 4).

All therapists demonstrated their ability to perform the mock‐WES‐RC and construct an appropriate treatment plan. As there were similarities between therapists about areas for improvement, generic feedback was provided to all (Supporting Information S1: File 8). Only six therapists’ WES‐RCs from their second participant were reviewed as planned, all of which were appropriate.

### 4.4 Discussion

The Workwell training course was successful in enabling therapists to conduct the WES‐RC and plan appropriate individualised treatment for a case study. Therapists found observing the WES‐RC in action particularly valuable and had the opportunity to practice with sample cases and discuss potential solutions with each other and the trainers. The Workwell Solutions Manual, used during training and the development of the mock WES‐RC treatment plan, was very useful to support therapists in identifying VR solutions. Therapists requested electronic versions of the WES‐RC and an online version of the Manual, which were provided during the trial.

The limitations included the limited formal mentoring and review of WES‐RCs from the therapists’ second participants. This was affected by the COVID‐19 pandemic, as the trial paused when only a quarter (33/124) of participants had completed treatment, most of whom were therapists’ first participants. Following the trial re‐start, changes had to be made to Workwell provision as many therapists had to provide this remotely (Ching et al. 2022). Ten therapists could no longer treat participants due to staff constraints. Therapists had difficulty identifying time within work hours for formal telephone mentoring because of increased pressure on services during the pandemic (e.g., staff redeployment, sickness absence). Therapists valued the information e‐mail discussion group and drop‐in sessions to request ideas for specific work problems.

## 5 Discussion

This component of the Workwell trial process evaluation identified the context in which Workwell would be delivered, and therapists’ need for VR training, particularly in conducting the work assessment and delivering Level 3 JRVR. The training course was modified based on feedback from the feasibility trial. Following training, therapists considered their VR knowledge improved and were more confident in identifying appropriate work solutions. Course content related to the WES‐RC was considered most useful, with the least being the pre‐course self‐study, as time was limited for study outside of the training course due to clinical commitments. The pre‐training self‐study pack was intended to facilitate therapists’ readiness for the course, particularly in work‐related activity analysis. The pack needs to be reduced to increase the likelihood of therapists engaging with this. To support uniform delivery of Workwell JRVR, we provided structured training in how to: conduct a work assessment (WES‐RC, plus the WES‐RC Manual), systematically identify and prioritise individual participant’s work problems, and plan individualised JRVR. This was supported by providing the Workwell Solutions Manual, working through case studies, and assessing therapists’ ability to complete a mock WES‐RC, with feedback. Mentoring support throughout the trial was planned but was impacted by the COVID‐19 pandemic. In the future, more post‐training support (mentoring) should be available to consolidate learning.

The availability of short courses to upskill therapists in VR is still limited. A list of resources and online and in‐person courses available is kept updated on the VRA website (VRA 2024). There are no short courses specifically for therapists on VR in RMD. Although the WORKWELL training course was targeted to IA, it could easily be modified to include other RMDs. The WES‐RC and WES‐RC Manual have been updated for use in the UK with people with IA (Hammond, O’Brien, et al. 2023; Hammond et al. 2023a, 2023b). A similar process would identify any further items essential for a range of RMDs, although likely few are needed. The course case studies could be adapted for other RMDs, as many symptoms associated with these are similar to IA (e.g., fatigue and pain). The content of the WORKWELL Solutions Manual remains appropriate as the goal of VR is to work collaboratively with the service user to identify solutions to work‐based problems from a functional perspective, as opposed to being diagnosis‐led. The practical workshop content would all be appropriate for other RMDs. The course has the potential to be adapted for other long‐term conditions.

## 6 Conclusion

This study identified that the updated Workwell JRVR training course improved participating therapists' knowledge about, confidence and ability to provide JRVR to employed people with IA, who have moderate‐to‐high risk of work disability (as identified using the RA‐WIS). Although developed for IA, it could be adapted for a wider range of RMDs with minimal modification. Given the time pressures on therapists, a virtual course with access to WES‐RC sample videos, sample cases, completed WES‐RCs and treatment notes, along with online access to the WES‐RC and Workwell Solutions Manual, could be beneficial for future development. A self‐help version of Workwell is available for service users, including a short work assessment (RA‐WIS), Workwell Solutions and interactive scenarios to put solutions into practice (Prior 2023 and Prior et al. 2024).

## Author Contributions

**Alison Hammond:** funding acquisition, conceptualisation, methodology, formal analysis, writing–original draft, writing–review and editing, resources. **Rachel O'Brien:** funding acquisition, conceptualisation, writing–original draft, writing–review and editing, resources. **Sarah Woodbridge:** funding acquisition, conceptualisation, writing–review and editing, resources. **Yeliz Prior:** funding acquisition, methodology, data collection, formal analysis, writing–review and editing. **Angela Ching:** project administration, formal analysis, writing–review and editing. **June Culley:** funding acquisition, conceptualisation, writing–review and editing. **Jennifer Parker:** project administration, formal analysis, writing–review and editing.

## Ethics Statement

This study was performed in line with the principles of the Declaration of Helsinki. Approval was granted by the University of Salford School of Health & Society Ethical Approval Panel (HSR1819‐010). Interview and training course participants provided informed, written consent.

## Conflicts of Interest

The authors declare no conflicts of interest.

## Supporting information

Supporting Information S1

## Data Availability

The data that support the findings of this study are available from the corresponding author on reasonable request.
