# Supplementary material for: Upskilling Rheumatology Occupational Therapists in Work Rehabilitation: An Evaluation of a Job Retention Vocational Rehabilitation Training Course (the Workwell Trial)
Source: Musculoskeletal Care. 2025 Feb 28;23(1):e70067. doi: 10.1002/msc.70067 (PMC11871416; doi:10.1002/msc.70067)
Supplement: Supplementary file 1 — Supporting Information S1 [file MSC-23-e70067-s001.pdf]

Supplementary Materials:

Upskilling rheumatology occupational therapists in work rehabilitation: an evaluation of a job retention vocational rehabilitation training course (the Workwell trial). Musculoskeletal Care (2025). Hammond A, O'Brien R, Woodbridge S, Prior Y, Ching A, Culley J, Parker J.

Page no	Supplementary File
2	Supplementary File 1: Thematic analysis: Workwell therapist interviews.
6	Supplementary File 2: Workwell Job Retention Vocational Rehabilitation
12	Supplementary File 3: Workwell pre-training self-study pack and Workwell therapist training course programme.
22	Supplementary File 4: Workwell Solutions Manual table of contents
35	Supplementary File 5: Evaluation: WORKWELL Training Course: Part 1 (Pre-training) Questionnaire
38	Supplementary File 6: Evaluation: WORKWELL Training Course: Part 2 (Post-training) Questionnaire.
45	Supplementary File 7: Mock WES-RC and mentoring evaluation form.
48	Supplementary File 8: Feedback to Therapists following the telephone mock-WES-RC.

Supplementary File 1: Thematic Analysis: Workwell Therapist interviews.

Pre-trial Work Service Interviews (n=9)		
**Main theme**	**Sub-themes**	**Illustrative data**
Referrals for work related problems	Clinical referrals	*"I mean, it's occasionally written on people's referrals.. Um, but more commonly, it's something that we pick up when we are chatting to people."* [OT8001]<br><br>*"So occasionally it will be mentioned, if it's been raised in their consultation, whether they're referred, if it's particularly discussed work being a problem, that might come up on the referral form."* [OT8002]<br><br>*"Sometimes the referrals comes through with work issues but not all of the time."* [OT8004]<br><br>*"They're, generally were referred from the, um, medical team. So the outpatient clinics so that could be doctor, registrar, nurse specialist."* [OT8006]
	Referrals from other agencies	*"we've had health and safety work advisors at work wanting a bit of support from the service. Um, return to work, we've had people that are getting close to maybe dismissal on medical grounds that maybe need a bit more support."* [OT8003]<br><br>"*They can self-refer because we do offer that, um, facility here."* [OT8006]
Work Assessment	Part of an initial OT assessment	"*…well under the initial assessment I ask around self-care productivity and leisure. And within a general discussion on those three areas I'd look at if they're in work.*." [OT8007]<br><br>*"When I do my initial assessment again work is on the assessment, so I'd ask about that, are they in employment? Are they having any problems?"*<br><br>*"Er, we don't use any, usually any standardised forms or anything like that, um, and we ask lots of relevant questions to the patient, um, looking at what type of role they've got, er, if they're in part of a team, all that sort of stuff."* [OT8005]<br><br>*"Okay, so usually we would discuss it as part of our initial ADL assessment and if patients identify*

Pre-trial Work Service Interviews (n=9)

**Main theme**	**Sub-themes**	**Illustrative data**
		any work problems then we will have a quite a brief discussion really, about ten minutes or so,.. But that would mostly be the limits of what we would ask for within our treatment." [OT002]
	Use of standardised work assessments	"*We also ..have the ankylosing spondylitis and the early arthritis clinic, actually complete the, um, RA-WIS and the AS-WIS so, again, if they score and it highlights that they need intervention .. then they get a referral generated to occupational therapy*."[OT8006]<br><br>*"Occasionally the consultants will use some screening tool but more than often not, their occupational therapist use our screening tool if that person's in work and that would be the RA Work Instability Scale which we, which we actually give to rheumatoid arthritis, psoriatic or in early inflammatory arthritis*."[OT8009]
Work Advice	Work interventions	"*Um, so, typically, sort of commonly at the minute, we might look at the ergonomic keyboard equipment that we've got, that quite commonly comes up. And we've got, um, a few different mice, err keyboards etcetera.*." [OT8001]<br><br>*"So we will give patients the NRAS booklets, if we feel they're relevant, so the booklets about wanting to stay in work and like I say, it'll be more within our usual care"* [OT8002]<br><br>*"So prior to doing the WORKWELL training the typical work-related advice I would give is probably, um, a discussion, um, it would be around, um, talking about the different resources that were available, normally the information booklet, the NRAS, the two booklets for employer, um, and the Versus Arthritis."* [OT8003]<br><br>"*I did used to use the NRAS, I want to work and the one for employers and, um, talk through, um, some of those issues, point them in the direction of places where they can get, um, ergonomic advice for work stations. Talk to people about modifying pens, looking at Dragon dictation. And I think I would, yeah, and fatigue management, those kind of side of things*." [OT8004]

Pre-trial Work Service Interviews (n=9)		
**Main theme**	**Sub-themes**	**Illustrative data**
		"… stress management is usually a biggie, and also looking at, um, quite often disclosure, um, the hidden symptoms of stuff..But it … but it spills over into work, and vice versa, work and home, and sometimes just by dealing with stuff at home the work side of things can improve as well." [OT8005]
	Duration of work interventions	*"About ten minutes, within a session, normally*." [OT8002]<br><br>"*Well, I'd probably say 10/15 minutes. Um, and then maybe that patient is going to be followed up so we would then say how did you get on doing the referral to your occupational health department, have there been any changes since that. So probably another half … so probably a half an hour in total*." [OT8003]<br><br>*"It, it depends on the, the individual because sometimes you're trying to guide them to make those life, err, changing decisions, um, and it's not always just a case of right, this'll be a good idea, why don't you try this?"* [OT8006]<br><br>"*About, about two or three sessions. So normally we um – because they get an hour each, an hour's assessment session and hour follow up, so um depending how complex they are, that I think I would average three sessions."* [OT8008]
	Referrals to other agencies	"*We've discussed with patients about the – looking at occupational health and in the workplace, because of us not normally having capacity to do workplace visits, so we will often recommend that if they've got that type of support, they do that um and access to work*" [OT8002]<br><br>"*Um, so I haven't referred directly. What I tend to do is give the patient the information, um, and let them decide whether they want to self-refer*."[OT8007]<br><br>"*Yeah, I've referred to Access for Work for a few people. Erm, I think that's the only thing – I've also er, Department of Work and Pensions, I've*

Pre-trial Work Service Interviews (n=9)

Main theme	Sub-themes	Illustrative data
		looked at, erm, erm referring patients on for, oh, I suppose they're the ones that have gone off sick actually. So I have used the local job centres as well…" [OT8008]<br><br>"*Um, a bit of signposting people, err, towards other support, err, systems, maybe Access for Work*." [OT8001]

Supplementary File 2:

WORKWELL

KEEPING PEOPLE WORKING

WORKWELL Job Retention Vocational Rehabilitation

1. **Work Self-help Information Pack**

Prior to the intervention, all participants receive this pack:

- a welcome letter, encouraging people to read the enclosed booklets, identify and discuss work problems and solutions with relatives, friends, and employers/line managers. For the intervention group, this additionally explained the Workwell therapist would help them with this process.
- a self-help flowchart, suggesting how to identify problems, resulting work difficulties and determine solutions to then implement and/or discuss with employers/ line managers.
- and four work self-help booklets including: a summary of the Equality Act (UK Government Equalities Office, 2010); how arthritis can affect work, benefits of staying in work, case studies of work issues people with arthritis have and how they resolved these (providing narratives and opportunities for modelling), common work problems and solutions, staying active, disclosure, the importance of discussing work issues with employers and colleagues, what "reasonable adjustments" employers could make, and how to get further help to manage at work: "I want to Work" (National Rheumatoid Arthritis Society, 2018); "Working with Arthritis" (Arthritis Care 2016/Versus Arthritis); "Work and Your Wellbeing" (Versus Arthritis 2018).

2. **WORKWELL Job Retention Vocational Rehabilitation** WORKWELL is based on that provided in the WORK-IA trial (Hammond et al, 2017) and trials conducted by Allaire et al (2003) and Macedo et al (2009). It is based on the biopsychosocial model and self-management approaches. In the appointment letter advance of the first meeting, it is emphasised that the more effort the patient can put into making changes at work, and to self-manage the effects of their arthritis, the more quickly results will be achieved; and asking participants to start reading the information pack sent to them. The intervention usually consists of between two to four meetings, with the first being a work-related interview, the UK Work Experience Survey-Rheumatic Conditions (WES-RC) (Hammond, Woodbridge, O'Brien & Grant, 2013). Each meeting generally lasts up to an hour. The first is held within four weeks of the participant receiving the self-help pack. Meetings are held at around four-week intervals (as agreed with the

participant), allowing time between meetings for participant and therapist to take appropriate actions. The number of meetings held depends on the nature of the work problems identified. Participants can be offered meetings earlier and later in the day to reduce impact on the working day. A telephone review is conducted six weeks after the last intervention meeting to check progress.

At the first meeting, the therapist uses the UK WES-RC. The WES-RC uses a biopsychosocial approach and includes a wide range of topics including: getting ready for and travel to/from work; workplace access; physical, mental and time job demands; relationships with people at work; environmental factors; company policies; job satisfaction and work life balance (Allaire & Keysor, 2009; Hammond et al, 2013; Hammond et al, 2019). The therapist works with the participant to:

- Identify work problems and specific barriers (physical, psychological, environmental (physical/social) and managerial) to overcome.
- Collaboratively agree three broad priority areas of work problems (e.g., workstation modification and work positioning; moving to/around the workplace), specifying problems within these

The therapist then recommends any applicable initial self-management advice tailored to the participant's needs, e.g., hand exercises, relaxation, sleep hygiene, fatigue and/or joint protection/ergonomic advice and provides appropriate booklets, e.g., Versus Arthritis' publications*: "Keep Moving," "Fatigue and Arthritis" and "Looking After Your Joints when you have arthritis," and/ or online resources, as applicable. These can be revisited in subsequent meetings to help put strategies into practice.

The therapist explains the benefits of action plans, and helps the participant to write an Action Plan, with a few initial, achievable SMART goals, to meet some immediate identified needs, and help the participant start taking appropriate actions to resolve work problems. Action plans can contribute to building self-efficacy. This meeting can take 1 to 1.5 hours, depending on the degree of difficulties the person is experiencing.

Participants are asked, before the next meeting, to complete an Activity Diary, for 24 hours on a typical workday, identifying for each 30-minute period: their main activity; any equipment, tools or materials used; any difficulties or discomfort doing the activity; whether they took a short rest; and to rate their level of pain and fatigue using a 0 (no) to 10 (severe) scale [modified with permission. This was modified with permission for the UK from Work Activity Diary (Backman C, Village L, Lacaille D, 2008). Participants are asked to reflect on problems identified in the WES-RC and activity diary and consider possible solutions, supported by selected reading in the self-help pack. If time, therapist and participant can start formulating solutions.

Between the first and second meeting, the therapist reviews the three priority problem areas identified in the WES-RC and develops a range of possible solutions to discuss with the participant in meeting 2. Developing solutions is supported by: using the Workwell Solutions Manual, which includes potential solutions and information resources linked to each section of the WES-RC; the therapists JRVR training and occupational therapy experience.

At this second meeting, the participant and therapist: review the Activity Diary to gain a better understanding of the person's workday; discuss the potential solutions therapist and participant propose and agree which solutions are workable; and the participant is supported to progressively make changes and put solutions into practice. AT the start of this, and any subsequent meetings, the therapist and participant review progress with action plans set in the previous meeting, collaboratively problem-solve, and the therapist continues supporting participants to write Action Plans and in resolving difficulties for themselves. Throughout the meetings, therapists emphasise the importance of participants taking responsibility for actions and to liaise with employers to request and obtain job accommodations.

A Treatment Standard Operating Procedure was provided to all therapists to remind them of treatment procedures.

WORKWELL JRVR is individualised to address the three prioritised problem work issues are jointly identified and the content can include any of the following strategies, as applicable and agreed with the participant:

a. applying ergonomic (e.g., altering work actions and work positioning, planning, prioritising, and scheduling), fatigue (e.g., pacing, microbreaks), stress (e.g., relaxation, mindfulness) management and work-productivity (e.g., time management, task prioritisation, delegation) approaches to the workplace.
b. recommendations for assistive technology/equipment adaptation, workplace/workstation modification, transport advice. Written information about equipment and resources is provided about these, as relevant to the participant's needs.
c. practical advice and support enabling participants to: discuss the pros and cons of disclosure and how to disclose their condition at work (if not already done so); and negotiate job modifications with employers/ line managers. This can include discussion of strategies, how to explain their condition's impact on work, and proactively make suggestions for addressing issues and role play, when applicable. Disclosure at work is an essential precursor for negotiating employer liaison.
d. explaining rights under the Equality Act 2010, how and why their condition meets being one applicable under the Equality Act, what are the range of "reasonable adjustments" they could request, and employers can make in the workplace. The facilities available to support employers making "reasonable adjustments," e.g., Access to Work

[https://www.gov.uk/access-to-work]; and how the participant can apply to Access to Work.

e. physical interventions, e.g., hand and upper limb exercise training, manual handling advice and training, recommendations for specific exercises and physical leisure activities to promote mobility, strength and stamina (e.g. walking, swimming, Tai Chi, Pilates, Yoga).

f. psychological support, through listening to and discussing work problems; encouraging ability and confidence in solving work problems, managing arthritis when working, and continuing working in future

g. advice on other activities of daily living difficulties affecting work ability

h. discussing relationships with managers and co-workers, and strategies for how to improve or manage these if presenting a barrier

i. discussing work-life balance and strategies to improve this and avoid role overload

j. to discuss, if necessary, about considering a change in job, role or career. Referral to a Disability Employment Advisor (DEA) at their local Jobcentre for advice and support in doing so, if the participant wishes

k. signposting and/ or referral, as relevant, to any other services identified as beneficial within and outside of the rheumatology or community multi-disciplinary team, such as ‘mainstream' occupational therapy (e.g. for splinting, activities of daily living training, self-management education), physiotherapy (e.g. for pain management, exercise therapy), podiatry (e.g. for insole or footwear provision), rheumatology nurse (e.g. advice on managing medications); state, employer and third sector work rehabilitation services as relevant (e.g. DEA; advice to contact their employer’s occupational health department; how to contact Access to Work); and to social work, local authority or community advisory services (e.g., Citizen’s Advice Bureau) for support related to housing, financial or welfare needs.

l. provision of relevant work and self-management advice booklets, on-line resources, and other information as appropriate

m. general advice on disease flare management and contacting the Rheumatology department quickly in a flare, for review of medication changes or steroid injection, if necessary, to reduce the risk of longer sickness absences

n. optional worksite visit: to conduct a workplace job assessment, and employer/ line manager liaison if required. The participant provides written consent to the work site visit and obtains permission in advance from the employer for this to occur. The therapist, as necessary, liaises in advance with the workplace to ensure any health and safety issues attending the worksite are explained and appropriate arrangements made. Practical solutions can be recommended and instituted during the visit.

o. Employer liaison: The extent of employer liaison is guided by the participant, as the therapist is working on behalf of the person rather than their employer. A letter can be provided for the employer/ line manager about the reasonable adjustments the participant needs. The content of the letter is agreed with the participant. This can be

supplemented with information for employers (NRAS: An Employer's Guide to Rheumatoid Arthritis: https://nras.org.uk/product/an-employers-guide-to-rheumatoid-arthritis/). The participant can provide the letter and/or booklet to their manager to support discussing their job accommodations needs in the workplace. If the participant wishes, a meeting can also be held between the therapist, participant, and their line manager to discuss relevant job accommodations, what is feasible and about referral to Access to Work for funding for equipment and/or other support, as applicable. The content of this meeting is discussed and agreed with the participant in advance. Alternately, employer liaison may be by telephone, with the participant's involvement.

p. A telephone review, six weeks after the final meeting, to discuss participants' progress implementing changes, check recommended job accommodations ae in place and if any further action is needed. This lasts up to 30 minutes.

q. The participant is provided with a discharge letter, summarising the issues, actions and recommendations provided in WORKWELL. This can be written to be suitable to provide to the employer/ line manager, if the participant wishes this.

<u>*Versus Arthritis downloads available from:</u>

Keep Moving:
https://www.versusarthritis.org/media/1310/keep-moving-information-booklet-with-poster.pdf

Looking After Your Joints:
https://www.versusarthritis.org/media/1271/looking-after-your-joints-information-booklet.pdf

Fatigue and Arthritis:
https://www.versusarthritis.org/media/1269/fatigue-and-arthritis-information-booklet.pdf

<u>References:</u>

Allaire SH, Li W, LaValley MP. (2003). Reduction of job loss in persons with rheumatic diseases receiving vocational rehabilitation: a randomized controlled trial. Arthritis & Rheumatism, 48(11), 3212-3218.

Allaire S, Keysor JJ. (2009). Development of a structured interview tool to help patients identify and solve rheumatic condition-related work barriers. Arthritis & Rheumatism, 61(7),988-995.

Backman C, Village L, Lacaille D, (2008). Activity Diary (personal communication, unpiublished).

Hammond A, Woodbridge S, O'Brien R, Grant M. (2013). The UK Work Experience Survey for persons with Rheumatic Conditions (UK WES-RC): University of Salford. https://salford-repository.worktribe.com/output/1430561/the-uk-work-experience-survey-for-persons-with-rheumatic-conditions-uk-wes-rc. [Updated in 2023 – see article reference list].

Hammond A, O'Brien R, Woodbridge S, Bradshaw L, Prior Y, Radford K, et al. (2017). Job retention vocational rehabilitation for employed people with inflammatory arthritis (WORK-IA): a feasibility randomized controlled trial. BMC Musculoskelet Disorders, 18(1):315.

Hammond A, Woodbridge S & O'Brien R. (2019). The UK-Work Environment Survey Manual v 3.1. University of Salford. https://salford-repository.worktribe.com/output/1366626/the-uk-work-experience-survey-for-persons-with-rheumatic-conditions-uk-wes-rc-manual-v31
[updated in 2023 – see article reference list].

Macedo AM, Oakley SP, Panayi GS, Kirkham BW. (2009). Functional and work outcomes improve in patients with rheumatoid arthritis who receive targeted, comprehensive occupational therapy. Arthritis & Rheumatism, 61(11),1522-1530.

Participant self-help work pack.

National Rheumatoid Arthritis Society (2018) " I want to work." https://nras.org.uk/wp-content/uploads/sites/2/woocommerce_uploads/2020/12/134579-I-Want-to-Work-Booklet-FINAL-2021-ukmknu.pdf (downloaded 14.8.2018).

Arthritis Care / Versus Arthritis (2016) "Working with Arthritis." (downloaded 14.8.2018)

Versus Arthritis (2018). "Work and Your Wellbeing" https://www.versusarthritis.org/about-arthritis/living-with-arthritis/work/ (downloaded 14.8.2018).

UK Government Equalities Office (2010), Equality Act 2010.

Supplementary File 3:

WORKWELL THERAPISTS TRAINING COURSE

The Training Course consists of three elements:

1. **Pre-course self- study**: approximately 1-day equivalent study. You do this in “bite size pieces” in the 2 months before the training course.

2. **Training course:** 2 days (programme included).

3. **Post-course:** approximately 1-day equivalent study within 1 month after the course. A “mock” telephone work interview for 1 hour with one of the trainers (within 2 weeks of the training course); write up your treatment plan and send to the trainers (within 2 weeks of interview). (See information). There will be a session during the training course explaining this activity.

Pre-course Self-study

1. **Introductory Reading – setting the scene:**

- "**Ergonomics for Therapists**" (Berg Rice, 2008), provides a background to ergonomics.
- "**Activity Analysis for Evaluation, Intervention Planning and Outcomes**" Thomas (2015): a reminder about the familiar core skill of activity analysis…and that the same process is used for work activities.

2. Activity Analysis: You Tube Links

Please watch the following jobs in these You Tube links. In the training course we have planned a session towards the end of Day 1 which will focus on your activity analysis of: **Russian Dolls; and Making Chocolate**. So please spend more time focused on these two clips. Please use the **Activity Analysis form** to guide you through the process of analysing these **two videos** (see end of pack).

- RUSSIAN DOLLS: https://www.youtube.com/watch?v=vtFC4_UaPzI
- MAKING CHOCOLATE: https://www.youtube.com/watch?v=3lBLqT0dDaU
- LAYING BLOCK PAVING: https://www.youtube.com/watch?v=wdzs9jeetQ0
- PROCESSING MEAT*: https://www.youtube.com/watch?v=1MRuCLXxqjs
- MAKING CHEESE: https://www.youtube.com/watch?v=l9lwiOnMVVU
- MAKING PIZZA: https://www.youtube.com/watch?v=eQK3YB4f38M

We have chosen a variety of occupations to show the range of factors you need to consider when analysing an individual's job. The clips are not too long: they range from a couple of minutes up to no more than 14 minutes. If you don't have time to view them all, focus on the two specified. These links have also been e-mailed to you, to make it easy to find them.

*Leave this one out if vegetarian/vegan.

3. WORKWELL Protocol sections:

Please read the sections relevant for WORKWELL therapists. (Summary in the pack).

4. **Optional:** *If you feel less familiar with different occupations /working environments*, you might like to view TV programmes such as:
   - **"How It's Made"** on Discovery; and Discovery Science Channel
   - **"Food Factory" & "How do they do it?"** both on Discovery Science Channel (weekday mornings repeated endlessly – record a few if you have access to these channels)
   - **"How do they do it": You Tube channel:** has short (3 -5 minute) videos about e.g. forklifts, making cricket balls … just Google to find them.
   - **Focus on: watching people working in TV programmes.**

WORKWELL THERAPISTS 2-DAY TRAINING PROGRAMME

Course Leaders: Rachel O'Brien, Sarah Woodbridge, Alison Hammond

**TIME**	**DAY 1:**
8.30 onwards	**REFRESHMENTS available on arrival**
9.00-9.20	Staff and therapists' introductions. Provision of Work Solutions Manual and training course materials.
9.20-10.00	Introduction to the WORKWELL trial: time scales; therapist roles; trial procedures
10.00-11.00	Introduction to the Work Structured Interview/ Assessment: the UK- Work Experience Survey- Rheumatic Conditions (WES-RC) and WES-RC Manual.<br>• Includes demonstration of WES-RC being conducted
**11.00- 11.30**	**REFRESHMENT BREAK**
11.30 – 1.00	Case Study discussions and using the WES-RC.<br>• Practical small group work: using the WES-RC with one of 4 case studies to: identify work issues and barriers; prioritise work problems; outline potential solutions (4 groups/ I case study each)
**1.00 – 2.00**	**BUFFET LUNCH**
**2.00-2.40**	Group case study feedback x 2 groups (each 20 minutes)<br>• Each group feeds back key points (5-10 minutes)<br>• Discussion with whole group/ trainers re problems identified and potential solutions
**2.40-2.45**	**COMFORT BREAK**
**2.45-3.30**	Group case study feedback x 2 groups (each 20 minutes)<br>• Each group feeds back key points (5-10 minutes)<br>Discussion with whole group/ SW/ ROB re problems identified and potential solutions
**3.30-3.50**	**REFRESHMENT BREAK**
3.50-4-20	Pre-study Activity Analysis: feedback on You Tube **Russian Dolls and Making Chocolate Activity Analysis**

4.20-4.45	Question Time: Activity Analysis; about today's sessions
4.45-5.00	Introduction to the **mock WES-RC telephone interview** (You will do this with Sarah or Rachel after the 2- day course)<br>**Summary and Close**

TIME	DAY 2: SESSION
**9.30-10.15**	**Practical Workshop 1**:<br>• Workstation assessment and seating
**10.15-11.00**	**Practical Workshop 2:**<br>• Upper limb strategies
**11.00-11.30**	**REFRESHMENT BREAK**
**11.30-12.15**	**Practical Workshop 3:**<br>• Environment
**12.15-12.45**	**Panel discussion with Patient Research Partners: June Culley and Peter Noskiw**<br>• Working with RA<br>• HR issues: seeking job modifications
**12.45-1.45**	**BUFFET LUNCH (with Patient Research Partners)**
**1.45-2.30**	**Practical Workshop 4:**<br>• Load Handling
**2.30-3.30**	**Practical Workshop 5: Disclosure; Rights and Employers**<br>• Discussing disclosure with service users; Rights and obligations under the Equality Act 2010/ Health & Safety At Work Act 1974; employer liaison; written communication with employers; work site visits.
**3.30-3.50**	**REFRESHMENT BREAK**
**3.50-4.50**	Reviewing the 4 case studies: 15 mins each.<br>Discussing potential solutions using Workshop Information and Work Solutions Manual.
**4.50-5.00**	**Final Questions and Close.**

Post-course Activity and Self-study.

Rachel or Sarah will arrange a time in advance with you to do the following:

- Within two weeks after the training course: conduct a **“mock” telephone WES-RC** with one of them. Rachel/Sarah will act as a client with work problems. You will have their case study information in advance. You will interview them using the WES-RC and work with the “client” to identify their priority problems. Record your findings and the priority problems on the WES-RC **during** the interview (as you would in practice). The interview will take up to 1 hour (as it would with a client).

Within 2 weeks of the mock WES-RCs, you will then:

- Using the course materials and the WORKWELL Work Solutions Manual, develop and write up a treatment plan for the client, including the work solutions for the client’s priority problems. Include the rationale for your choices.
- Send a copy of the completed WES-RC and Treatment Plan to Rachel/ Sarah (as applicable) within the next 2 weeks.

About 2 weeks later:

- Rachel/ Sarah will provide you with feedback.

Successfully completing the pre-course self-study, the training course, the mock WES-RC and your treatment plan means you will be awarded with a WORKWELL Training Certificate and you will be prepared to deliver the WORKWELL intervention.

- *If you experience difficulty with the mock WES-RC and Treatment Plan you can do this again with either Rachel or Sarah.*

Ongoing support: Mentoring

Once you start delivering WORKWELL, Rachel or Sarah will arrange to conduct a mentoring session with you. They can discuss and review your WES-RC and treatment plan for a participant you are treating. You can also obtain advice by e-mail and arrange to discuss any issues by telephone with them. Additionally, we will have group online meetings and an e-mail discussion group through which you can discuss treatment issues with other WORKWELL therapists and the mentors.

ACTIVITY ANALYSIS FORM

**PERFORMANCE SKILLS**	
**A Motor skills**	
Posture	
Mobility	
Coordination	
Strength & effort	
Energy	

**B Process skills**	
Energy	
Knowledge	
Temporal organisation	
Organising space and objects	
Adaptation	

**C Communication/ Interaction skills**	
Physicality	
Information exchange	
Relations	
**<u>CLIENT FACTORS</u>**	
Mental functions	
Sensory functions and pain	
Neuromuscular and movement related functions	

**<u>3 MOST IMPORTANT FACTORS OF YOUR ACTIVTY ANALYSIS</u>**	**1.**<br><br>**2.**<br><br>**3.**
**<u>STRATEGIES FOR ADAPTATION</u>**	1.<br><br>2.<br><br>3.
**COMMENTS**	

Supplementary File 4:

WORKWELL

KEEPING PEOPLE WORKING

WORKWELL SOLUTIONS MANUAL: Introduction and Table of Contents

Welcome to the **WORKWELL Solutions Manual**. This Manual is designed to be used with the Work Experience Survey- Rheumatic Conditions (WES-RC). When you identify key work barriers in the WES-RC, look up the relevant section in this WORKWELL Solutions Manual to find handy tips, strategies, practical advice and solutions.
Combine the relevant work solutions and advice with your clinical expertise, knowledge of self-management for musculoskeletal conditions and therapeutic skills to develop the individualised WORKWELL intervention meeting your client's priority work-related needs.

Let us know your suggestions, practical solutions and ideas to add to the Manual.

All the best in developing and delivering the WORKWELL intervention.

Alison Hammond, Sarah Woodbridge, Rachel O'Brien

Table of Contents

**File Tab 1**		
**Section 1: WORKWELL documentation**		
**1**	Summary of the WORKWELL Intervention and Work Self-Help information pack	
**2**	The UK Work Experience Survey- Rheumatic Conditions	
**3**	The UK Work Experience Survey- Rheumatic Conditions Manual	
**4**	WORKWELL Therapy Appointment Letter v2	
**5**	WORKWELL Activity Diary	
**6**	WORKWELL Action Plan	
**7**	WORKWELL Treatment Record: Part 1 and Part 2	
**8**	**The Participant Work Self-Help Information Pack**:<br>• Cover letters to WORKWELL and Control group participants.<br>• Finding Solutions flowchart;<br>• NRAS “I want to Work” (2018);<br>• https://www.nras.org.uk/publications/i-want-to-work<br>• Arthritis Care “Working with Arthritis” (2016); https://www.versusarthritis.org/media/1422/working-with-arthritis-booklet.pdf<br>• Versus Arthritis (Arthritis Research UK web information “Working with Arthritis and Joint Pain” (2018);<br>• https://www.versusarthritis.org/about-arthritis/living-with-arthritis/work/<br>• UK Gov Equalities Office “The Equality Act: what do I need to know” (2010)<br>• https://assets.publishing.service.gov.uk/government/uploads/system/uploads/attachment_data/file/85028/vcs-service-providers.pdf	

**File Tab 2**		
**UK WES-RC Section 2: Getting to Work & the Work Environment**		
**Travelling to and from work, or for work:**		
1	Public transport	
2	Driving<br>• RIDC Motoring with Arthritis<br>• https://www.ridc.org.uk/content/motoring-arthritis<br>• Posturite Car Ergonomics checklist<br>• https://www.posturite.co.uk/media/pdf-downloads/Car-Ergonomics.pdf	

**File Tab 3**		
**UK-WES-RC Section 3: Workplace Access**		
**Getting into and Around your Place of Work**		
1	Access	
**Using Workplace Facilities**		
2	Toilet<br>• Welfare at work – Health and Safety Executive (**HSE)** Guidance for employers on welfare provisions http://www.hse.gov.uk/pubns/indg293.htm	
3	Refreshments	
4	Emergency Exit<br>• Personal Evacuation Plan	

**File Tab 4**		
**UK-WES-RC Section 4: Vocational Rehabilitation Solutions: Completing Activities**		
**Physical Job Demands**		
1	Ergonomics and Human Factors at Work<br>• http://www.hse.gov.uk/pubns/indg90.pdf<br><br>MSD Hazards & Solutions<br>https://www.uwo.ca/hr/form_doc/health_safety/doc/ergo/msd_hazards_solutions.pdf	
2	Lifting strategies<br><br>Manual Handling:<br>• HSE Manual Handling at work: a brief guide http://www.hse.gov.uk/pubns/indg143.pdf<br>• HSE Manual handling assessment chart (the MAC Tool)<br>• http://www.hse.gov.uk/pubns/indg383.pdf<br>• HSE MAC score sheet<br>• https://www.hse.gov.uk/forms/mac/macscore.pdf<br>• ALSO follow the weblink to:<br>• http://www.hse.gov.uk/msd/mac/ for interactive score sheets and further information<br>• HSE Making the best use of lifting and handling aids<br>• http://www.hse.gov.uk/pubns/indg398.pdf	
3	Pushing and pulling	
4	Ladders<br>• HSE Safe use of ladders and step ladders http://www.hse.gov.uk/pubns/indg455.htm	

**File Tab 5**		
**WES-RC Section 4: Completing Activities (continued)**		
**Physical Job Demands: Work Station Evaluation, Computer Use, Handling Objects**		
**1**	Work Station Assessment:<br>• HSE VDU Work Station Checklist http://www.hse.gov.uk/pubns/ck1.pdf<br>• HSE Working with Display Screen Equipment<br>• http://www.hse.gov.uk/pubns/indg36.pdf<br>• Ability Net: “Workstation Ergonomics ”<br>• https://www.abilitynet.org.uk/factsheets/ergonomics-and-computing	
**2**	Seating<br>• Selecting a chair, issues for consideration<br>• National seating suppliers<br>• Osmond ergonomics Posture Guidance<br>• https://www.ergonomics.co.uk/res/Posture_Guidance1.pdf<br>• HSE Seating at Work<br>• http://www.hse.gov.uk/pUbns/priced/hsg57.pdf	
**3**	Desk<br>• RSIA Repetitive Strain Awareness leaflet: correct work station set-up	
**4**	Computer monitor<br>• My computer My way – Abilitynet<br>• https://mcmw.abilitynet.org.uk/mcmw	
**5**	Keyboard<br>• Osmond Ergonomics range of inputting devices – keyboards https://www.ergonomics.co.uk/ergonomic-keyboards.html<br>• Ability Net Rheumatoid Arthritis and Computing	

	• https://www.abilitynet.org.uk/factsheets/rheumatoid-arthritis-and-computing<br>• Ability Net: Keyboard and Mouse Alternatives and Adaptations<br>• https://www.abilitynet.org.uk/factsheets/keyboard-and-mouse-alternatives-and-adaptations-disabled-people<br>• Abilitynet: Controlling your computer tablet or phone with your voice<br>• https://www.abilitynet.org.uk/factsheets/controlling-computer-tablet-or-smartphone-your-voice	
**6**	Mouse<br>• Selecting a mouse<br>• Osmond Ergonomics: Selection of Ergonomic Mice: https://www.ergonomics.co.uk/iqs/rp.1/ergonomic_mice_and_trackballs.html<br>• Ability Net: Keyboard and Mouse Alternatives and Adaptations<br>• https://www.abilitynet.org.uk/factsheets/keyboard-and-mouse-alternatives-and-adaptations-disabled-people	
**7**	Wrist Rest<br>• Wrist rest selection summary	
**8**	Document holder / writing slope<br>• Osmond Ergonomics range of document holders and slopes<br>• https://www.ergonomics.co.uk/document-holders.html	
**9**	Laptop<br>• Osmond Ergonomics range of laptop stands<br>• https://www.ergonomics.co.uk/ergonomic-laptop-stands.html	
**10**	Osmond Portable Electronic Devices: Agile Working Posture Guidance	

	• https://www.ergonomics.co.uk/res/Agile_Working_Posture_Guide<br>• Osmond ergonomics range of tablet smartphone stands<br>• https://www.ergonomics.co.uk/ipad-tablet-accessories.html	
**11**	Writing<br>• Writing aids	
**12**	Telephone	
**13**	Osmond Ergonomics Stretching Exercises for Sedentary Workers<br>• https://www.ergonomics.co.uk/res/Stretching_Excercises1.pdf	

**File Tab 6**		
**UK-WES-RC Section 4: Completing Activities (continued)**		
**Mental, Time, Energy and Emotional Demands**		
**1**	Mental Health Issues and Work	
**2**	Working together to reduce stress at work<br>http://www.hse.gov.uk/pubns/indg424.pdf	
**3**	HSE Stress Indicator Tool<br>http://www.hse.gov.uk/stress/assets/docs/indicatortool.pdf	
**4**	HSE Line Managers Resource: a practical guide to managing and supporting people with mental health issues in the workplace<br>http://www.hse.gov.uk/stress/assets/docs/manage-mental-health.pdf	
**5**	HSE Return to Work questionnaire<br>http://www.hse.gov.uk/stress/assets/docs/returntowork.pdf	

**File Tab 7**		
**UK-WES-RC: Section 5 Relationships with People at Work; Disclosure**		
1	Fit For Work: Talking to your employer about your health<br><br>https://fitforwork.org/blog/talking-to-your-employer-about-your-health/	
**2**	Disability Discrimination: key points for the workplace<br><br>http://www.acas.org.uk/media/pdf/s/k/Disability_discrim_keypoints_workplace_Nov.pdf	

**File Tab 8**		
**UK-WES-RC Section 6: Environmental Factors and Company Policies; Benefits & Legislation**		
**Environmental Factors**		
**1**	Lighting	
**2**	Temperature	
**3**	Noise	
**4**	Flooring<br>• Floor protection<br>• Specialist Flooring	
**Company Policies**		
**1**	Policies	
**2**	Employer Guide: Access to Work	
**3**	Benefits<br>The Benefits System: A Short Guide for GPs<br>https://assets.publishing.service.gov.uk/government/uploads/system/uploads/attachment_data/file/691502/the-benefits-system-a-short-guide-for-gps.pdf	

**File Tab 9**		
**UK-WES-RC Section 7: Job, Career and Home Life**		
**1**	Work Life Balance and Job Satisfaction<br>• Mayo Clinic: Work Life Balance: Tips to maintain control<br>• https://www.mayoclinic.org/healthy-lifestyle/adult-health/in-depth/work-life-balance/art-20048134	

**File Tab 10 -**		
**Supporting documents**		
10	IOSH Occupational health management in the workplace https://www.iosh.co.uk/Books-and-resources/Occupational-health-management-in-the-workplace.aspx	
11	Health and Safety Executive: Managing sickness absence and return to work http://www.hse.gov.uk/pUbns/priced/hsg249.pdf	
12	Getting the most out of the Fit Note: GP Guidance https://www.gov.uk/government/publications/fit-note-guidance-for-gps<br>The Fit Note: a guide for patients and employees https://www.gov.uk/government/publications/the-fit-note-a-guide-for-patients-and-employees	
13	HSE The Health & Safety Toolbox http://www.hse.gov.uk/pubns/priced/hsg268.pdf	
14	Royal College of OT; “Work Matters: vocational navigation for occupational therapy staff https://www.rcot.co.uk/practice-resources/rcot-publications/downloads/work-matters	

**File Tab 15**		
	**Your resources and notes**	

© Alison Hammond, Sarah Woodbridge, Rachel O’Brien, (2018).

Supplementary File 5: Evaluation: WORKWELL Training Course: Part 1 (Pre-training) Questionnaire

**Your Name**	
**Hospital Name**	
**Your Band?**<br>(*delete as applicable)*	5 6 7 8
**Your Profession**<br>(*delete as applicable)*	OT PT
**Years working as a therapist?**	
**Years' experience in Rheumatology?**	
**Therapy qualification/ year awarded**	
**Post graduate qualification/s year(s)**	

We would like to know your views about your ability to provide work rehabilitation at present. Please could you indicate your opinion by **circling** the relevant option below.

	**Very Limited**	**Limited**	**Moderate**	**Good**	**Excellent**
**your knowledge of VR**	☐	☐	☐	☐	☐
**your knowledge of the VR process**	☐	☐	☐	☐	☐
**your knowledge of VR strategies**	☐	☐	☐	☐	☐
**your knowledge of relevant legislation and policy**	☐	☐	☐	☐	☐
**your confidence completing a work assessment**	☐	☐	☐	☐	☐
**your confidence in identifying work solutions and strategies**	☐	☐	☐	☐	☐

The following questions ask about your feelings about using new types of therapy, interventions, or treatments. "Manualized therapy" (questions 6 and 7) refers to an intervention that has specific guidelines and/or components that are outlined in a manual and/ or that are being followed in a structured/ predetermined way. **WORKWELL** is one such example but please consider others.

Please indicate the extent to which you agree with each item using the below scale by circling the appropriate number below

	Not at all	To a Slight Extent	To a Moderate Extent	To a Great Extent	To a Very Great Extent
1 I like to use new types of therapy / interventions to help my clients	☐	☐	☐	☐	☐
2 I am willing to try new types of therapy / interventions even if I have to follow a treatment manual	☐	☐	☐	☐	☐
3 I know better than academic researchers how to care for my patients	☐	☐	☐	☐	☐
4 I am willing to use new and different types of therapy/interventions developed by researchers	☐	☐	☐	☐	☐
5 Research based treatments/ interventions are not clinically useful	☐	☐	☐	☐	☐
6 Clinical experience is more important than using manualised therapy/ interventions	☐	☐	☐	☐	☐
7 I would not use manualised therapy/ interventions	☐	☐	☐	☐	☐
8 I would try a new therapy/ intervention even if it was very different from what I am used to doing	☐	☐	☐	☐	☐

**For the questions 9 - 15, If you receive/d training in a therapy or intervention that was new to you, how likely would you be to adopt it if: ?**					
	**Not at all**	**To a Slight Extent**	**To a Moderate Extent**	**To a Great Extent**	**To a Very Great Extent**
9 It was intuitively appealing to you	☐	☐	☐	☐	☐
10 It “made sense” to you?	☐	☐	☐	☐	☐
11 It was required by your immediate supervisor?	☐	☐	☐	☐	☐
12 It was required by your Therapy service?	☐	☐	☐	☐	☐
13 It was required by your NHS Trust?	☐	☐	☐	☐	☐
14 It was being used by your colleagues who were happy with it?	☐	☐	☐	☐	☐
15 You felt you had enough training to use it correctly?	☐	☐	☐	☐	☐

THANK YOU FOR TAKING THE TIME TO COMPLETE THIS.

Supplementary File 6: Evaluation: WORKWELL Training Course: Part 2 (Post-training) Questionnaire

**Your Name**	
**Hospital Name**	

We would like your views on the **relevance** of the training/study you have completed.

Please rate the relevance of:

Pre-training study pack:

	Not at all relevant	Limited relevance	Moderate Relevance	Very relevant	Extremely relevant
Book chapter: Ergonomics and Therapy: an Introduction (Berg Rice)	☐	☐	☐	☐	☐
Book Chapter: Activity Analysis for Evaluation (Thomas)	☐	☐	☐	☐	☐
Activity Analysis using You Tube videos (e.g. Russian Dolls; chocolate making etc)	☐	☐	☐	☐	☐
Protocol sections (including Background; WORKWELL)	☐	☐	☐	☐	☐

Training Course Day 1:

	Not at all relevant	Limited relevance	Moderate Relevance	Very relevant	Extremely relevant
Introduction to WORKWELL trial	☐	☐	☐	☐	☐
Introduction to WES-RC (including Sarah/Rachel's role play of WES-RC interview – "Sally the hairdresser"	☐	☐	☐	☐	☐
Case studies with the WES-RC (4 cases)	☐	☐	☐	☐	☐
Group case study feedback	☐	☐	☐	☐	☐
Review of pre-training study activity analysis (You Tube videos)	☐	☐	☐	☐	☐
Question time: Activity Analysis	☐	☐	☐	☐	☐
Introduction to mock telephone WES-RC activity with your mentor	☐	☐	☐	☐	☐

Training Course Day 2:

	Not at all relevant	Limited relevance	Moderate Relevance	Very relevant	Extremely relevant
Workshop 1: Workstation assessment	☐	☐	☐	☐	☐
Workshop 2: Upper limb strategies	☐	☐	☐	☐	☐
Workshop 3: Environment	☐	☐	☐	☐	☐
Discussion: Working with RA (Patient Partners)	☐	☐	☐	☐	☐
HR Issues	☐	☐	☐	☐	☐
Workshop 4: Load Handling	☐	☐	☐	☐	☐
Workshop 5: Disclosure, Rights and Employers	☐	☐	☐	☐	☐
Case Studies Review: Resource Manual and Solutions	☐	☐	☐	☐	☐
Avoiding Control Group Contamination & What You can do with the Control Group	☐	☐	☐	☐	☐

Post- training

	Not at all relevant	Limited relevance	Moderate Relevance	Very relevant	Extremely relevant
Telephone Mock WES-RC with mentor	☐	☐	☐	☐	☐
• Prioritising problems	☐	☐	☐	☐	☐
• Planning your intervention	☐	☐	☐	☐	☐
Feedback on Telephone WES-RC	☐	☐	☐	☐	☐

We would like to know your views about your ability to provide work rehabilitation now. Please could you indicate your opinion by **circling** the relevant option below.

	Very Limited	Limited	Moderate	Good	Excellent
**your knowledge of VR**	☐	☐	☐	☐	☐
**your knowledge of the VR process**	☐	☐	☐	☐	☐
**your knowledge of VR strategies**	☐	☐	☐	☐	☐
**your knowledge of relevant legislation and policy**	☐	☐	☐	☐	☐
**your confidence completing a work assessment**	☐	☐	☐	☐	☐
**your confidence in identifying work solutions and strategies**	☐	☐	☐	☐	☐

To help us develop this programme further please could you identify 3 things:

You gained the most benefit from:

You did not feel were useful:

Please give us your suggestions on how we can improve the training programme; and deliver it differently.

Are there any other practical activities you would have wanted to be included?

YES **NO**

If yes, please list:

Do you have any comment about the amount of time you have spent on training for this trial?
N.B. Training is currently 4 days (ie 2 days contact and up to 2 days self-directed study including pre-reading and activity analysis; the mock telephone WES-RC with your mentor and formulating your treatment plan) .

How many days do you consider the training should take?

Any other comments?

Finally, the following questions ask about your feelings about using new types of therapy, interventions, or treatments. "Manualized therapy" (questions 6 and 7) refers to an intervention that has specific guidelines and/or components that are outlined in a manual and/ or that are being followed in a structured/ predetermined way. **WORKWELL** is one such example but please consider others.

Please indicate the extent to which you agree with each item using the below scale by circling the appropriate number below

	Not at all	To a Slight Extent	To a Moderate Extent	To a Great Extent	To a Very Great Extent
1 I like to use new types of therapy / interventions to help my clients	☐	☐	☐	☐	☐
2 I am willing to try new types of therapy / interventions even if I have to follow a treatment manual	☐	☐	☐	☐	☐
3 I know better than academic researchers how to care for my patients	☐	☐	☐	☐	☐
4 I am willing to use new and different types of therapy/interventions developed by researchers	☐	☐	☐	☐	☐
5 Research based treatments/ interventions are not clinically useful	☐	☐	☐	☐	☐
6 Clinical experience is more important than using manualised therapy/ interventions	☐	☐	☐	☐	☐
7 I would not use manualised therapy/ interventions	☐	☐	☐	☐	☐
8 I would try a new therapy/ intervention even if it was very different from what I am used to doing	☐	☐	☐	☐	☐

**For the questions 9 - 15, If you receive/d training in a therapy or intervention that was new to you, how likely would you be to adopt it if: ?**					
	**Not at all**	**To a Slight Extent**	**To a Moderate Extent**	**To a Great Extent**	**To a Very Great Extent**
9 It was intuitively appealing to you	☐	☐	☐	☐	☐
10 It "made sense" to you?	☐	☐	☐	☐	☐
11 It was required by your immediate supervisor?	☐	☐	☐	☐	☐
12 It was required by your Therapy service?	☐	☐	☐	☐	☐
13 It was required by your NHS Trust?	☐	☐	☐	☐	☐
14 It was being used by your colleagues who were happy with it?	☐	☐	☐	☐	☐
15 You felt you had enough training to use it correctly?	☐	☐	☐	☐	☐

THANK YOU FOR TAKING THE TIME TO COMPLETE THIS.

Supplementary File 7:

WORKWELL THERAPIST MOCK WES-RC /MENTORING FORM

**Mentor :**	
**Therapist :**	
**Case study/ Participant PIN:**	
**Date:**	

**WES-RC section**	**Good (1) Satisfactory (2) Poor (3)**	**Comments** *(for mock-WE-RC include comments on interview process)*
Section 1: Demographic & health		
Work history		
Action points:		• •
Section 2: Getting ready & travel		
Action points:		• •

Section 3:<br>Access & facilities:		
Action points:		•<br>•
Section 4:<br>Job activities		
Action points:		•<br>•
Section 5:<br>Relationships		
Action points:		•<br>•
Section 6:<br>Environment & policies:		
Action points:		•<br>•
Section 7:<br>Work/ life balance		
Action points:		•<br>•

**SECTION 8: PROBLEMS IDENTIFIED AND SOLUTIONS OFFERED**<br>*(Do problems follow on from assessment and solutions from problems).*		
Problem 1		
Solution(s) to problem 1		•<br>•
Problem 2		
Solution(s) to problem 2		•<br>•
Problem 3		
Solution(s) to problem 3		•<br>•
Action points:		•<br>•
**Overall and Any other comments:**		

Supplementary File 8:

Feedback to Therapists following the Mock-WES-RC.

- Following your great job at interviewing Ruth or Carla [*role played characters by the trainers/ assessors*], remember it will be easier and more natural for you to use your initial interview technique when filling out the WES-RC, rather than going through the form section by section. This will mean that it is more conversational and less stilted but may result in you moving backwards and forward through the WES-RC. The only possible negative of adopting this method is that you may not ask some questions routinely which are in the WES-RC. In order to ensure this doesn't happen we recommend that you familiarise yourself with the WES-RC before treating participants. If you don't usually ask about lighting in the workplace, for example, make a mental note to ask about this before each assessment.

- When you meet the patient, introduce yourself and thank them for being in the trial. Introduce the WES-RC and let them know that this is the assessment you are completing in the research.

- We appreciate your feedback regarding the mock WES-RC interview and acknowledge that it was an artificial situation, which made it more difficult for some of you to feel you gained the information needed. We are confident that in a face-to-face situation with a patient you would **all** be able to gather the information required.

- Throughout the assessment it is really useful to keep going back to the main issues after each section, and recap with the patient to validate your conclusions. This will also help the patient to identify their principal problems as well.

- Try to avoid using jargon. As health professionals we have a lot of knowledge which the patients may not understand or be familiar with. For instance, during the interviews many of you talked about fatigue, but some patients may not understand what that means. Avoid terms like pacing and energy conservation, as they won't know what

these terms mean. Think about real world alternatives and use their wording: if they say tired, adopt that; for pacing say something like, ways to help you balance out the everyday things you do; and for energy conservation say something like, ways to make doing things less tiring.

- Throughout the assessment, think whether there are repeated patterns of movement across different work tasks which are impacting on function and pain. For example, are they gripping different tools, or using repeated wrist deviation across different aspects of their job?

- As the assessment progresses, if it becomes apparent that you are able to provide a solution at the same time as the assessment e.g., if discussing shift patterns and the patient identifies they need to change shift patterns, then discuss this immediately after completing the assessment and plan. Start VR straight away. Aim to reduce the number of follow-up appointments needed.

- If at any point you don't think you covered something in sufficient detail, feel free to go back to the issue and gather more information. It's OK to do this.

- When identifying the barriers make sure they relate specifically to **work,** e.g., knee pain is not a work barrier, **but standing for prolonged periods is** relevant to work and would exacerbate knee pain.

- At the end, we thought about your immediate action plan post-interview and considered you need to give the patient something to go away with to signal treatment has started. We don't want you to get them back a few weeks later to do more assessments, so think about whether anything else you need to know could be incorporated into the WES-RC if it only takes a couple of minutes e.g., observing hand movements.

- It is appropriate and useful to: check all patients received the Versus Arthritis and NRAS booklets [*in the patient information pack posted out prior to the appointment*], and to

ask them to read these (and provide them if they did not receive); and ask **all** patients to complete the activity diary This will start to provide some education and also help them to start to see whether there are any triggers during their workday for their work problems. Also identify relevant pages within the booklets for patients to read, to make it meaningful and relevant for each individual.

- Remember, another assessment is not a solution. Try to get all the necessary information during the work interview.

- A person can receive, for example, joint protection education or a splint as part of JRVR, if it is specifically related to work, as these are work interventions.
